# Children’s mobile-gaming preferences, online risks, and mental health

**DOI:** 10.1371/journal.pone.0278290

**Published:** 2022-12-01

**Authors:** Chun-Yin Hou, Ru Rutherford, Hsi Chang, Fong-Ching Chang, Liu Shumei, Chiung-Hui Chiu, Ping-Hung Chen, Jeng-Tung Chiang, Nae-Fang Miao, Hung-Yi Chuang, Chie-Chien Tseng

**Affiliations:** 1 Department of Family Medicine, Taipei City Hospital, Zhongxiao Branch, Taipei, Taiwan; 2 Department of Health Promotion and Health Education, National Taiwan Normal University, Taipei, Taiwan; 3 Department of Pediatrics, School of Medicine, College of Medicine, Taipei Medical University, Taipei, Taiwan; 4 Department of Preschool Education, Jing Hengyi College of Education, Hangzhou Normal University, Hangzhou, China; 5 Graduate Institute of Information and Computer Education, National Taiwan Normal University, Taipei, Taiwan; 6 The Graduate Institute of Mass Communication, National Taiwan Normal University, Taipei, Taiwan; 7 Department of Statistics, National Chengchi University, Taipei, Taiwan; 8 Post-Baccalaureate Program in Nursing, College of Nursing, Taipei Medical University, Taipei, Taiwan; 9 Department of Public Health, Kaohsiung Medical University, Kaohsiung, Taiwan; Hiroshima International University: Hiroshima Kokusai Daigaku, JAPAN

## Abstract

This study examined the relationships between children’s mobile gaming preferences, online risks, and mental health. Data were obtained from a sample of 2,702 third and fourth grade students from 16 elementary schools in Taiwan and 9 schools in China. A self-administered questionnaire was used. The mental state of the children who participated in the study was assessed using the Strengths and Difficulties Questionnaire (SDQ), while mobile gaming addiction was assessed using the short form of the Internet Gaming Disorders Scale (IGDS9-SF). The results showed that about 54% of children played mobile games with others (multi-player), while 31% played mobile games alone, and 15% did not play mobile games. Multiple logistic regression results indicated that behaviors such as participating in multi-player games, playing violent games, a poor parent-child relationship, and living in a rural area were associated with a greater risk of mobile gaming addiction. Involvement in multi-player games, playing violent games, mobile gaming addiction, and exposure to mobile violence/pornography were associated with greater risks of cyber aggression/victimization. Multiple regression results showed that being a multi-player, playing violent games, mobile gaming addiction, exposure to violence/pornography, exposure to cyber aggression/victimization, and having a poor parent-child relationship were associated with emotional and behavioral problems.

## Introduction

With the proliferation of mobile devices, online gaming has become a part of children’s daily lives, and the average duration of their online sessions has increased. A 2019 UK national survey showed that two-thirds of 8–11 y/o children play online games [1]. As the usage of these electronic devices has increased, so has both the incidence and the scope of adverse effects [2]. Previous studies have associated an adolescent’s online gaming behaviors with online risks such as Internet gaming addiction [3], exposure to violence/pornography [4], and cyber aggression/victimization [5]. Children and adolescents with an Internet gaming addiction were more likely to experience poorer sleep quality [6], depression [7], social anxiety [8], somatic disorders [9], a poorer quality of life [10], and lower school performance [11].

The American Psychiatric Association included Internet Gaming Disorders in Section III (disorders requiring further investigation) of the fifth edition of the Diagnostic and Statistical Manual of Mental Disorders (DSM-5) in 2013, while the World Health Organization included Gaming Disorders in the 11th revision of the International Classification of Diseases (ICD-11) in 2018 [12]. These publications used "internet gaming disorder," "online gaming addiction," and "problem gaming" as interchangeable terms. Asia has some of the world’s largest concentrations of populations and has a high and rising prevalence of Internet and smartphone use disorders [13]. Studies have estimated that the prevalence of Internet Gaming Disorders among children and adolescents averages 1–9% [14], and that the prevalence of online gaming addiction among adolescents in Asian countries (10–15%) is higher than that in Western countries (1–10%) [15]. In Taiwan, about one-fifth of children and adolescents engage in problematic mobile gaming [16]. In addition, studies have indicated that game genres such as massive multi-player online role-playing games [17], fighting games [18], and multi-player online battle arenas [19] were associated with Internet gaming addiction among both children and adolescents.

Physical aggression and cyber aggression/victimization among children and adolescents represents an emerging public health problem that is becoming prevalent among Asian adolescents [20, 21]. In Australia, the prevalence of traditional bullying victimization among adolescents was 25% and perpetration was 12%, while cyberbullying victimization and perpetration were 7 and 3%, respectively [22]. In Hong Kong, Mainland China, and Taiwan, 33.0, 23.8, and 31.7% of adolescents, respectively, reported experiencing cyberbullying in the past semester, while 20.4, 7.7, and 20.6% of adolescents reported engaging in cyberbullying against others [21]. A review study found that exposure to violent video games was a causal risk factor for increases in aggressive behavior, aggressive cognition, and aggressive affects with decreases in empathy and prosocial behaviors [23]. Studies associated exposure to violent online games with cyberbullying [5] and physical aggression [24], and children who spent more time playing games were more likely to be involved in cyberbullying [25, 26]. Adolescents who had cyber aggression/victimization experiences were more likely to have emotional and psychosomatic problems, and tended to have difficulties with social adjustment [27–29].

As children spend greater amounts of time playing mobile games, parents and experts worry that children might develop mobile gaming addictions and encounter online risks that could cause psychological distress [1, 14]. China has the world’s largest population, and China’s one-child policy and culturally inflected social control experiences could have had an impact on children’s internet use and online risks [30]. Prior studies conducted in China and Taiwan have indicated that children and adolescents who reside in rural areas spend more time playing online games and are more vulnerable to online risks such as cyber victimization compared with their urban counterparts [31, 32]. Despite studies have associated young adult and adolescent problematic gaming behaviors and internet addictions with online risks and psychological distress [33–35], only a limited amount of research has explored the relationships between children’s gaming preferences and online risks, and exploring the links to the possible psychological impact. Despite existing evidence of the links between problematic gaming behaviors, Internet addictions, online risks, and psychological distress among adolescents and young adults [33–35], only a limited amount of research has explored the relationships between children’s gaming preferences and online risks, and exploring the links to the possible psychological impact. Unlike previous studies that mainly recruited adolescents, this study involved third- and fourth-grade children between 9–11 years of age. In addition, this study compared children participants from urban and rural areas in Taiwan and China to examine urban-rural differences in different socio-economic backgrounds and explore relationships between children’s gaming preferences (multi-player games, single-player games, and non-players), online risks (mobile gaming addiction, mobile violence/pornography exposure, and cyber aggression/victimization experiences), and mental health.

## Methods

### Participants

The participants were third- and fourth-grade primary school students from the Taipei area and Pingtung County in Taiwan and the Shouguang urban and rural areas in Shandong Province, China. The rural/urban classification is based on the population size, population density, and degree of urbanization of a given country [36–38]. In 2018, the number of primary schools in Taipei City and Pingtung County in Taiwan was 153 and 168, respectively, according to data from the Taiwan Ministry of Education, while the number of primary schools in the Shouguang urban and rural areas in Shandong Province, China, was 7 and 92, respectively, according to data from the Shouguang Ministry of Education. Based on the sampling frame, a probability-proportionate-to-size sampling method was used to systematically draw a random sample of schools. Three-to-four school classes were randomly selected from each school. This study invited 30 sample schools, and only 5 sample schools refused to participate in the survey. Of the 25 sample schools that participated in this study, 9 are in the Taipei area (urban), 7 reside in Pingtung County (rural), Taiwan, 3 are located in urban Shouguang, and 6 are in rural Shouguang, Shandong Province, China. In addition, this study sent letters to 2,910 parents requesting their consent to have their primary school children participate in the survey. About 95% of parents agreed that their children could participate. In 2018, there were 2,702 third- and fourth-grade students aged 9–11 years who completed the questionnaire. The questionnaires were delivered to students in their classrooms by trained interviewers. Students were assured that their information would be protected and anonymous. When a student had any questions during the survey, the trained interviewers answered the questions and provided assistance. The study was reviewed by the Research Ethics Committee of National Taiwan Normal University (REC No. 201802HS004).

### Measures

#### Mobile gaming preferences

Mobile gaming preferences were measured with two items. Children were asked the following: (1) Do you play games on mobile phones or tablets? The response option for each item was either “no” or “yes”. If children answered “no” for playing games on mobile phones or tablets, then they were categorized as a “non-player”. If children answered “yes”, then they were asked a further question: (2) Do you play games alone or with other players on mobile phones or tablets? The response options included “playing games alone” and “playing games with other players”. If children answered “playing games alone” then they were categorized as a “single-player”. If children answered “playing games with other players”, then they were categorized as “multi-players”.

#### Mobile gaming time

Mobile gaming time was computed using children’s reports of hours per week spent playing games on smartphones and tablets. The weekly use time of mobile gaming was calculated based on the following two questions: (1) During the past week, how much time did you spend playing games on smartphones or tablets per weekday (Monday to Friday)?; and, (2) During the past week, how much time did you spend playing games on smartphones or tablets per weekend day (Saturday and Sunday)?

#### Mobile gaming addiction

Mobile gaming addiction was measured with 9 items adapted from the short form of the Internet Gaming Disorders Scale (IGDS9-SF), [39] which was developed according to 9 criteria of Internet Gaming Disorder, as suggested by the American Psychiatric Association. A review study found that the IGDS9-SF has great psychometric properties across different language versions [40]. Sample questions were as follows: “Do you feel more irritability, anxiety or even sadness when you try to either reduce or stop your gaming activity?”; “Do you feel the need to spend increasing amounts of time engaged in gaming in order to achieve satisfaction or pleasure?”; and, “Have you continued your gaming activity despite knowing it was causing problems between you and other people?”. The response option was either “yes” or “no”. As suggested by the DSM-5 the provision of the IGD diagnosis required at least five out of nine criteria [41]. If students reported “yes” for five items or more, they were categorized as having mobile gaming addiction.

#### Mobile violent game playing

Mobile violent game playing was measured with one item. Children were asked the degree of violent content in the mobile games they played frequently. The response options included “no violence”, “moderate violence”, and “severe violence”. If children answered “moderate violence” or “severe violence”, they were coded as playing violent mobile games.

#### Mobile violence/pornography exposure

Mobile violence and pornography exposure was assessed with two items. Children were asked the following questions: (1) In the past year have you seen violent messages or images on smartphones or tablets?; and, (2) In the past year have you seen pornographic messages or images on smartphones or tablets? The response options for each item included “never”, “sometimes”, and “always”. If children answered “sometimes” or “always”, they were coded as having mobile violence/pornography exposure.

#### Cyber aggression/victimization

Children’s cyber victimization was measured with four items from the past year to which children responded with either yes or no: (1) someone made rude comments about me online; (2) someone posted embarrassing or nude photos of me online; (3) someone spread rumors to hurt me online; and, (4) I was bullied by my classmates or friends online. If children answered “yes” for each item, they were coded as cyber victimization. In addition, children’s cyber aggression was measured with three items from the past year to which children responded with either yes or no: (1) I made rude comments to someone online; (2) I spread rumors about someone online; and, (3) I bullied other peers online. If children answered “yes” for each item, they were coded as cyber aggression.

#### Parent-child relationship

Parent-child relationships were measured using 2 items. Children were asked the following questions: “How did you get along with your mother?” and, “How did you get along with your father?” Response options included “not good” (scoring 1), “general” (scoring 2), and “good” (scoring 3).

#### The Strengths and Difficulties Questionnaire (SDQ)

The SDQ is a widely used and validated instrument to measure psychological adjustment among children and adolescents [42]. The Chinese version of SDQ [43] has demonstrated acceptable levels of reliability and validity and was used in this study. The SDQ consists of five subscales with five items each: emotional symptoms, conduct problems, hyperactivity-inattention, peer problems, and prosocial behaviors. Each item was rated on a 3-point scale: not true (scoring 0), somewhat true (scoring 1), and certainly true (scoring 2). The total difficulties score refers to the sum of the scores on four subscales: emotional symptoms, conduct problems, hyperactivity-inattention, and peer problems. The Cronbach’s alpha of the total difficulties scale for the sample was 0.74, while the Cronbach’s alpha of the prosocial behaviors scale was 0.75.

#### Child characteristics

Child characteristics obtained in this study included children’s reports of gender (girl vs. boy) and grade (fourth vs. third).

### Statistical analysis

SAS software was used to perform the statistical analysis. Percentages and means were calculated for all variables. A series of multiple logistic regressions were conducted to examine relationships between children’s demographic background, mobile gaming preferences, and online risks (mobile gaming addiction, mobile violence/pornography exposure, and cyber aggression/victimization). In addition, multiple regression analyses were performed to examine relationships between children’s mobile gaming preferences, online risks, and psychosocial adjustment (SDQ attributes). This study included children who did not play mobile games (15%) as a reference group of mobile gaming preferences in the analysis.

## Results

### Children’s mobile gaming preferences and online risks

Children who participated in this research included 1,284 boys (47.5%) and 1,418 girls (52.5%). Of the participants, 1,534 students (56.6%) were from Taiwan, while 1,177 (43.4%) were from China. In addition, 1,135 students (41.9%) lived in urban areas, while 1,576 (58.1%) lived in rural areas. There were no significant differences between urban and rural areas in Taiwan and in China with respect to participants’ gender and grade (Table 1).

**Table 1:** Children’s demographic characteristics and online risks by region and urban/rural areas.

	Taiwan				China				Region
Variables	All %	Urban %	Rural %	P value	All %	Urban %	Rural %	P value	P value
Gender									
Girl	48.4	48.5	48.4	0.966	45.9	43.6	46.6	0.387	0.189
Boy	51.6	51.5	51.6		54.1	56.4	53.4		
Grade									
3^rd^ grade	43.7	43.5	44.0	0.853	45.0	42.4	45.7	0.348	0.522
4^th^ grade	56.3	56.5	56.0		55.0	57.6	54.3		
Mobile gaming preference									
Non-player	12.1	13.0	10.8	0.011*	19.8	23.3	18.8	0.190	<0.001***
Single-player	32.9	35.3	29.8		28.8	25.7	29.7		
Multi-player	55.0	51.7	59.4		51.4	51.0	51.5		
Mobile game addiction									
Yes	16.9	14.1	20.6	0.001****	6.1	4.2	6.7	0.131	<0.001***
No	83.1	85.9	79.4		93.9	95.8	93.3		
Mobile violent game playing									
Yes	24.5	22.4	27.4	0.022*	21.0	21.2	20.9	0.927	0.029*
No	75.5	77.6	72.6		79.0	78.8	79.1		
Mobile violence exposure									
Yes	24.5	22.0	27.8	0.007***	35.6	31.6	36.8	0.118	<0.001***
No	75.5	78.0	72.2		64.4	68.4	63.2		
Mobile pornography exposure									
Yes	18.1	16.7	20.0	0.093	15.2	12.2	16.1	0.119	0.042*
No	81.9	83.3	80.0		84.8	87.8	83.9		
Cyber victimization									
Yes	8.2	7.0	9.8	0.047*	14.2	10.6	15.3	0.059	<0.001***
No	91.8	93.0	90.2		85.8	89.4	84.7		
Cyber aggression									
Yes	3.6	2.2	5.5	0.001***	5.1	3.8	5.5	0.264	0.055
No	96.4	97.8	94.5		94.9	96.2	94.5		

Note. Chi-squared tests were conducted.

Taiwan urban n = 876; Taiwan rural n = 658; China urban n = 259; China rural n = 918.

Regional comparison results show that Taiwan children have significantly higher rates of multi-players, gaming addictions, mobile violent game playing, and mobile pornography exposure, while children from China have significantly higher rates of mobile violence exposure and cyber victimization. In Taiwan, urban-rural comparison results show that by comparison with urban children, rural children have significantly higher rates of multi-players and online risks. In China, urban-rural comparison results showed no significance.

*p<0.05

**p<0.01

***p<0.001

A total of 1,446 children (53.5%) played online games with other players on smartphones or tablets (multiple-player), while 841 children (31.1%) played games alone on smartphones or tablets (single-player) and 415 children (15.4%) didn’t play games (non-players) (Table 3). Children who were mobile gaming multi-players spent 8.82 hours playing games per week, while children who were single-players spent 5.17 hours playing games per week (Table 4).

About 12.2% of children had mobile gaming addiction. In addition, 27.4% of children had been exposed to violence on smartphones or tablets, while 16.6% of children had been exposed to pornography on smartphones or tablets. Moreover, 10.9% of children had experienced cyber victimization and 4.3% reported cyber aggression (Table 3).

### Children’s online risks by regions and urban/rural areas

When comparing children’s online risks by region, children from Taiwan spent 6.74 hours playing games per week, while children from China spent 4.66 hours playing games per week (Table 2). Children from Taiwan had a higher proportion of multi-player use (55%) than children from China (51.4%) (*X*^2^ (2, N = 2711) = 31.11, *p* < .001), while children from Taiwan also had a higher proportion of mobile gaming addictions (16.9%) than children from China (6.1%) (*X*^2^ (1, N = 2754) = 72.66, *p* < .001). Children from Taiwan had a higher proportion of mobile violent game playing (24.6%) than children from China (21%) (*X*^2^ (1, N = 2754) = 4.79, *p* = 0.0286), while children from Taiwan also had a higher proportion of exposure to mobile pornography (18.2%) than children from China (15.2%) (*X*^2^ (1, N = 2754) = 72.66, *p* < .001). In addition, children from China had a higher proportion of exposure to mobile violence (35.6%) than children from Taiwan (24.5%) (*X*^2^ (2, N = 2747) = 40.44, *p* < .001), while children from China also had a higher proportion of cyber victimization (14.2%) than children from Taiwan (8.2%) (*X*^2^ (1, N = 2748) = 25.49, *p* < .001) (Table 1).

**Table 2:** Children’s mobile gaming time and SDQ by gaming region and urban/rural areas.

	Taiwan				China				Region
Variables	All Mean	Urban Mean	Rural Mean	P value	All Mean	Urban Mean	Rural Mean	P value	P value
Mobile gaming time (hr/week)	6.74	5.63	8.22	<0.001***	4.66	5.31	4.47	0.323	<0.001***
Parent-child relationship	2.75	2.77	2.74	0.257	2.87	2.84	2.88	0.043	<0.001***
SDQ attributes									
Total difficulties	11.46	11.01	12.05	<0.001***	11.62	11.65	11.61	0.929	0.478
Prosocial behaviors	6.93	7.14	6.67	<0.001***	7.40	7.33	7.43	0.522	<0.001***

Note. t tests were conducted.

Taiwan urban n = 876; Taiwan rural n = 658; China urban n = 259; China rural n = 918.

Regional comparison results show that Taiwan children spend more time on mobile gaming, while children from China have better parent-child relationships and better prosocial behavior. In Taiwan, urban-rural comparison results show that rural children spend greater amounts of time on mobile gaming, have higher total difficulties scores, and have lower prosocial behavior scores than urban children. In China, by comparison with urban children, rural children have better parent-child relationships.

*p<0.05

**p<0.01

***p<0.001

When comparing children’s online risks between urban and rural areas in Taiwan, children from rural area had a higher proportion of multi-player use (59.4%) than children from urban areas (51.7%) (*X*^2^ (2, N = 1534) = 8.99, *p* = 0.011), while children from rural areas also had a higher proportion of mobile gaming addictions (20.6%) than children from urban areas (14.1%) (*X*^2^ (1, N = 1564) = 11.67, *p* < .001). Children from rural areas in Taiwan had a higher proportion of mobile violent game playing (27.4%) than children from urban areas (22.4%) (*X*^2^ (1, N = 1564) = 5.23, *p* = .002), while children from rural areas also had a higher proportion of exposure to mobile violence (27.4%) than children from urban areas (21.9%) (*X*^2^ (1, N = 1560) = 7.28, *p* = .007). In addition, children from rural areas in Taiwan had a higher proportion of cyber victimization (9.8%) than children from urban areas (7%) (*X*^2^ (1, N = 1561) = 3.93, *p* = .047), while children from rural areas also had a higher proportion of cyber aggression (5.5%) than children from urban areas (2.3%) (*X*^2^ (1, N = 1563) = 11.38, *p* = .007). In addition, there were no significantly differences in online risks between children from rural and urban areas in China (Table 1).

### Children’s SDQ attributes by regions and urban/rural areas

Comparing children’s SDQ attributes by region, children from China had greater prosocial behaviors score than children from Taiwan. Comparing children’s SDQ attributes between urban and rural areas in Taiwan, children from rural areas had higher total difficulties scores than children from urban areas, while children from urban areas had higher prosocial behaviors score than children from rural areas. In China, children from rural areas had slightly better parent-child relationship than children from urban areas, while there were no significant differences in SDQ attributes between children from rural and urban areas (Table 2).

### Children’s online risks by mobile gaming preferences

When comparing children’s online risks by gaming preferences, multi-players had a higher proportion of mobile gaming addictions (17.5%) than single-players (9.0%) and non-players (0%) (*X*^2^ (2, N = 2711) = 104.75, *p* < .001). Multi-players had a higher proportion of exposure to mobile violence (35.7%) than single-players (24.4%) and non-players (17.1%) (*X*^2^ (2, N = 2706) = 68.36, *p* < .001), while multi-players also had a higher proportion of exposure to mobile pornography (19.9%) than single-players (16.3%) and non-players (8.2%) (*X*^2^ (2, N = 2701) = 31.70, *p* < .001). In addition, multi-players had a higher proportion of cyber victimization (15.3%) than single-players (7.1%) and non-players (3.4%) (*X*^2^ (2, N = 2708) = 65.30, *p* < .001), while multi-players also had a higher proportion of cyber aggression (6.4%) than single-players (2.6%) and non-players (0.2%) (*X*^2^ (2, N = 2709) = 38.61, *p* < .001) (Table 3).

**Table 3:** Children’s demographic characteristics and online risks by gaming preferences.

	Total		Non-player		Single-player		Multi-player		
Variables	N	%	n	%	n	%	n	%	P value
Gender									<0.001***
Girl	1418	52.5	272	65.5	466	55.4	546	37.8	
Boy	1284	47.5	143	34.5	375	44.6	900	62.2	
Grade									0.197
3^rd^ grade	1200	44.3	199	47.6	380	45.0	621	42.9	
4^th^ grade	1511	55.7	219	52.4	464	55.0	828	57.1	
Resident location									0.165
Urban	1135	41.9	175	41.9	375	44.4	585	40.4	
Rural	1576	58.1	243	58.1	469	55.6	864	59.6	
Region									< .001***
Taiwan	1534	56.6	185	44.3	505	59.8	844	58.3	
China	1177	43.4	233	55.7	339	40.2	605	41.8	
Mobile gaming addiction									< .001***
Yes	330	12.2	0	0.0	76	9.0	254	17.5	
No	2381	87.8	418	100.0	768	91.0	1195	82.5	
Mobile violent game playing									< .001***
Yes	623	23.1	0	0.0	123	14.6	502	34.7	
No	2079	76.9	415	100.0	718	85.4	944	65.3	
Mobile violence exposure									< .001***
Yes	741	27.4	71	17.1	205	24.4	517	35.7	
No	1965	72.6	345	82.9	636	75.6	932	64.3	
Mobile pornography exposure									< .001***
Yes	448	16.6	34	8.2	137	16.3	287	19.9	
No	2252	83.4	382	91.8	703	83.7	1158	80.1	
Cyber victimization									< .001***
Yes	295	10.9	14	3.4	60	7.1	221	15.3	
No	2413	89.1	404	96.7	782	92.9	1227	84.7	
Cyber aggression									< .001***
Yes	116	4.3	1	0.2	22	2.6	93	6.4	
No	2593	95.7	417	99.8	821	97.4	1355	93.6	

Note. Chi-squared tests were conducted.

Mobile gaming preferences comparison results show that multi-players have higher rates of online risks compared with that of either single-players or non-players.

*p<0.05 **p<0.01 ***p<0.001

### Children’s SDQ attributes by mobile gaming preferences

Comparing children’s SDQ attributes by their preferences of gaming, multi-players had higher total difficulties score (Mean = 12.15) than single-players (Mean = 11.12) and non-players (Mean = 10.11). In contrast, non-players had higher scores for prosocial behaviors than multi-players (Table 4).

**Table 4:** Children’s mobile gaming time and SDQ by gaming preferences.

	Non-player		Single-player		Multi-player			
	Mean	SD	Mean	SD	Mean	SD	P value	Post-hoc tests
Mobile gaming time (hr/week)	0.0	0.0	5.17	9.31	8.82	13.41	< .001***	M>S>N
Parent-child relationship	2.83	0.34	2.82	0.37	2.79	0.39	0.174	
SDQ attributes								
Total difficulties	10.11	5.65	11.12	5.63	12.15	5.41	< .001***	M>S>N
Prosocial behaviors	7.47	2.30	7.23	2.35	7.00	2.31	0.001***	N>M

Note. ANOVA tests were conducted. Post-hoc tests were analyzed by Scheffe’s test.

Non-player n = 415; Single-player n = 841; Multi-player n = 1446.

N, non-player; S, single-player; M, multi-player.

Mobile gaming preferences comparison results show that multi-players spend a greater amount of time on mobile gaming and have higher scores for total difficulties and lower scores for prosocial behaviors by comparison with either single-players or non-players.

*p<0.05 **p<0.01 ***p<0.001

### Factors related to children’s online risks

Multiple logistic regression results revealed that factors related to children’s mobile gaming addictions included being a multi-player, playing violent games, having a poor parent-child relationship, and rural residence in Taiwan. In addition, factors related to children’s mobile violence exposure included being in the 4th grade, being a multi-player, playing violent games, having mobile gaming addictions, and urban residence in China, while factors related to children’s mobile pornography exposure included being a multi-player, playing violent games, a poor parent-child relationship, and having mobile gaming addictions (Table 5).

**Table 5:** Factors related to children’s online risks.

	Mobile gaming addiction	Mobile violence exposure	Mobile pornography exposure	Cyber victimization	Cyber aggression
	β	β	β	β	β
Intercept	-1.13	-2.30***	-0.87	-3.37***	-3.88***
Gender (boy = 1, girl = 0)	0.17	0.10	-0.03	0.45**	0.50*
Grade (4th grade = 1, 3th grade = 0)	-0.09	0.34***	-0.13	0.17	0.15
Mobile gaming preferences (multi-player = 1, single-/non-player = 0)	0.85***	0.46***	0.20	0.58***	0.66*
Mobile violent game playing (yes = 1, no = 0)	1.09***	0.60***	0.46***	0.60***	0.78***
Parent-child relationship (1–3)	-0.92***	-0.06	-0.28*	-0.19	-0.64**
Resident location (rural = 1, urban = 0)	0.39**	0.23*	0.16	0.20	0.45
Region (Taiwan = 1, China = 0)	1.14***	-0.63***	0.12	-0.79***	-0.48*
Mobile gaming addiction (yes = 1, no = 0)		0.61***	0.74***	0.80***	0.93***
Mobile violence exposure (yes = 1, no = 0)				0.83***	0.81***
Mobile pornography exposure (yes = 1, no = 0)				0.66***	0.87***

Note. Multiple logistic regression models were conducted.

Mobile gaming addiction model n = 2681; Mobile violence model n = 2677; Mobile pornography model n = 2672; Cyber victimization model n = 2668; Cyber aggression model n = 2669.

The results associate multiplayers with greater levels of online risk.

*p<0.05

**p<0.01

***p<0.001

Multiple logistic regression results indicated that factors related to children’s cyber victimization included being male, being involved in multi-player gaming, playing violent games, having mobile gaming addictions, mobile violence/pornography exposure, and residence in China. In addition, factors related to children’s cyber aggression included multi-player gaming, playing violent games, mobile gaming addictions, mobile violence/pornography exposure, a poor parent-child relationship, and residence in China (Table 5).

### Relationships of children’s gaming preferences, online risks and SDQ attributes

Multiple regression results indicated that a greater total difficulties score was associated with the following characteristics: a 3^rd^ grade level, multi-player gaming, mobile gaming addictions, mobile violence/pornography exposure, cyber aggression/victimization, a poor parent-child relationship, and being a resident of China. In contrast, the following characteristics were associated with a greater prosocial behaviors score: a 4th grade level, female, no mobile gaming addictions, a better parent-child relationship, and residing in an urban area of China (Table 6).

**Table 6:** Factors related to children’s SDQ.

	Total difficulties	Prosocial behaviors
	β	β
Intercept	18.49***	3.83***
Gender (boy = 1, girl = 0)	0.06	-0.40***
Grade (4th grade = 1, 3th grade = 0)	-0.67**	0.29***
Mobile gaming preferences (multi-player = 1, single-/non-player = 0)	0.44*	0.02
Mobile violent game playing (yes = 1, no = 0)	0.86**	-0.30**
Mobile gaming addiction (yes = 1, no = 0)	2.91***	-0.90***
Mobile violence exposure (yes = 1, no = 0)	0.74**	0.02
Mobile pornography exposure (yes = 1, no = 0)	0.80**	-0.07
Cyber victimization (yes = 1, no = 0)	1.06**	0.08
Cyber aggression (yes = 1, no = 0)	2.23***	-0.43
Parent-child relationship (1–3)	-2.08***	1.06***
Resident location (rural = 1, urban = 0)	0.26	-0.19*
Region (Taiwan = 1, China = 0)	-0.52*	-0.30**

Note. Multiple regression models were conducted.

Total difficulties model n = 2619; Prosocial behaviors model n = 2652.

The results indicate that multiplayers and online risk is associated with higher total difficulties scores, while online risk is also associated with lower scores for prosocial behaviors.

*p<0.05

**p<0.01

***p<0.001

## Discussion

This study showed that multi-players spent more time playing mobile games and had a higher proportion of mobile gaming addictions than single-players. These results were compatible with those of previous studies [44, 45], which have established a higher risk of addiction for online multi-player games as well as increased difficulties for players to quit due to characteristics such as competitiveness and cooperativeness that are characteristic of online multi-player games. Prior studies also found that adolescents playing some game genres such as online shooting games and multi-player online role-playing games that involve massive numbers of players were positively associated with Internet game addictions [19, 46–48]. During the past few decades, online gaming marketing has increased dramatically, and more children have had problems with gaming. A cohort prior study found that exposure to online gaming during preschool years was associated with IGD risks in adolescence [49]. Governments could regulate online gaming marketing and implement age-restriction policies to protect children from playing online games at younger ages and developing gaming addictions.

In addition, the present study found that compared with non-players and single-players, multi-players encounter a greater number of online risks and problematic SDQ behaviors. One of the possible reasons could be the high potential risk for addiction and problematic behaviors for online multi-players. This study was consistent with prior studies that found adolescents who had an Internet gaming addiction were more likely to have emotional and psychosocial problems [8, 47, 50]. Studies also found that children’s and adolescents’ Internet gaming addiction could be associated with hyperactivity-inattention [3, 51, 52]. Another study found that the risk for problematic gaming was associated with a younger age of gaming initiation and suggested ways for parents to actively prevent this [53]. Parents could cautiously consider which age to allow children to play online games and own smartphones, while parents could also set rules about what games children can play and whether they can play games with others.

Another important result gleaned from this study was the risk of mobile violence/pornography exposure. This study showed that more than one-fourth of children reported mobile violence exposure and one-seventh reported exposure to mobile pornography. Since online game advertisements are compulsorily broadcasted under the current cyber environment, it is not difficult to imagine how frequent an advertisement could be viewed unintentionally through the Internet, particularly when these advertisements contain violence and/or pornographic messages, which could adversely affect children who are still developing both physically and mentally. It is reasonable to predict that the exposure frequency and amount of violence and pornography encountered by multi-players would be remarkably higher than that seen by single-players. Although exposure to violence was known to increase the risk of attention-deficit/hyperactivity disorder (ADHD) [54, 55], this study showed that “online” violence exposure and playing violent games could be as harmful as “actual” violent experiences for school-aged children who develop ADHD. Since children can easily declare adult age online to play violent games, governments could develop strategies to prevent children’s exposure to violent games.

In addition, this study found that more than one-tenth of children had cyber aggression/victimization experiences. Children who reported playing violent mobile games, having mobile gaming addictions, and being exposed to violence/pornography were more likely to experience cyber aggression/victimization, and these factors were positively associated with emotional and behavioral problems. Prior research has revealed that violent media exposure causes an increase in aggressive thoughts, angry feelings, physiologic arousal, hostile appraisals, and aggressive behavior [4]. Children’s exposure to online violence appeared to increase the risk of aggression [56]. Prior studies also found children’s and adolescent’s Internet gaming addictions were related to cyber aggression/victimization [57]. Since children’s school bullying often extended from cyberbullying, school bullying prevention programs could include cyberbullying prevention strategies to enhance children’s digital competence. Parents and teachers could also try to understand children’s online activities and persons they contact online. A study [58] found that increasing student engagement in schools could be a strategy to reduce students’ school bullying and cyberbullying victimization.

This study found that multi-players had higher total difficulties score. Previous studies [59, 60] have claimed a close association between conduct disorders and both bullying and bully victimization. Multi-players were at high risk for mobile gaming addictions, which increased the frequency of exposure to mobile violence and pornography and often was the cause of a pathological formation of conduct disorders and cyber aggression/victimization. Prior studies [61, 62] have indicated that conduct disorders were considered a precursor of anti-social behavior in adulthood, which underscores the importance of catching the early signs of online gaming addiction among children.

Moreover, this study associated a poor parent-child relationship with children’s mobile gaming addiction, mobile pornography exposure, cyber aggression, and emotional and behavioral problems. Prior studies also associated a poor parent-child relationship with Internet gaming addiction [63, 64], while low levels of family affection were associated with greater emotional and behavioral problems in children [65]. Parents could develop good relationships with children and understand their mobile gaming behaviors in order to mediate their online gaming time, content, and gaming preferences. The American Academy of Pediatrics has recommended that parents become involved with their child’s use of media and must ensure that their children have ample media-free time and access to nongaming play [14].

In addition, this study found that children in Taiwan spent more time playing mobile games and have higher levels of mobile gaming addiction, while children in China reported higher levels of exposure to violence, and cyberbullying experiences. These differences might reflect different cultures, media regulation, Internet policies, education and child policies between Taiwan and China. In China, obsessive online gaming behavior among youth is viewed as a national issue of public health and social control [30], while gaming addiction is commonly addressed as an individual syndrome of self-control in Taiwan [66]. Future studies could further explore whether access to different mobile devices, Internet resources, gaming patterns, digital literacy, educational pressure, one-child policies, and parental mediation in different regions might influence children’s online activities, online risks, and psychological distress. Since massive multiplayer online role-playing games were the most addictive [17, 67], more studies are needed to examine the causes and impact within different cultural contexts.

Moreover, this study found that children who reside in rural areas spend more time playing mobile games and experience more online risks than children who reside in urban areas in Taiwan. These results were consistent with prior study [31] that showed children who reside in rural areas spend more time online and are more vulnerable to online risks. However, this study found no differences between urban and rural areas in China, which is a result that differs from that of prior study [32]. The reason may be due to the sample schools in China being located in the same province and possibly having similar living resources. How to balance digital opportunities and risks for children, particularly in rural areas, becomes challenging. The Taiwan government has established broadband in rural areas to expand Internet access and enhance digital opportunities. However, children from rural areas who usually have lower levels of parental mediation and less educational arrangements are more likely to spend more time online and engaged in online risks. Governments could provide resources for schools and communities to enhance the digital literacy of both parents and children and increase media-free educational opportunities for children to prevent online risks.

### Limitations

This study had some limitations. First, this was a cross-sectional study, which limits the information that can be used to infer causality. Second, because of the children’s young ages and the differences in their regional backgrounds, they could have perceived different meanings for the SDQ attributes in the questionnaire. This could explain the low reliability of SDQ subscales in this study, while studies [68–70] also found low reliability for some SDQ subscales and interpretation of the subscales difficult for certain ethnic groups. Thus, we did not present the results of SDQ subscales. Third, many words in pornographic messages might not be understood by third- and fourth-grade school children and may have a different impact. Fourth, in the present study we focused on exploring children’s mobile gaming addictions, while there could be a discrepancy between mobile gaming addictions and online gaming additions. However, our study found that most children reported using smartphones to go online instead of computers. Fifth, this study compared urban and rural factors based on official data from Taiwan and China, while the definition of urban and rural areas might differ in different regions. Thus, we conducted an urban/rural comparison in each region and controlled for the urban/rural area and region variables in the multivariate analysis. Finally, the instrument of mobile gaming addiction was adapted from IGD-SF9. The pilot test indicated that 3^rd^ and 4^th^ grade school children had difficulties in answering the 5 point-Likert scale, thus we dichotomized the response. According to DSM-5 suggestion we chose the cut-off point of 5 out of 9 criteria to estimate the prevalence of IGD, while a review study found that there remained problems regarding using some instruments with different cut-off points to compare IGD prevalence across different culture regions and across different demographic background participants [71]. Future studies could develop a more appropriate instrument to assess children’s Internet gaming disorders and employed models and analysis of the causal inferences of explored relationships. A related approach could be longitudinal examinations to better understand the longitudinal and/or reciprocal relationships of mobile gaming preferences, online risks, and psychological/behavioral problems while exploring the transitional effects of mobile gaming on the psychosocial adjustment of children and adolescents.

### Implications

This study found that children who were multi-players, played violent games, had a mobile gaming addiction, and had exposure to mobile violence/pornography were more likely to be involved in cyber aggression/victimization and to have emotional and behavioral problems. To prevent online risks and psychological distress, it is suggested that parents could understand children’s online activities and have discussions with children regarding what games they can play, when they can play, whether they can play games online, whether they can play games with other players. During the COVID-19 pandemic period, studies have indicated that school children were more likely to have psychological distress, spend more time online, and engage in problematic internet-related activities [72, 73]. Studies have encouraged parents and teachers to educate children about using smartphones and social media safely [74], to understand and monitor children’s internet using habits and activities [75], to incorporate social support into cyberbullying intervention [29], and to facilitate positive activities to prevent psychological distress among schoolchildren particular during COVID-19 [72].

## Conclusions

The findings of the present study indicated that more than half of the children surveyed played mobile games with other players. Children who were multi-players had a higher proportion of online risk such as mobile gaming addiction and exposure to mobile violence/pornography and cyber aggression/victimization compared with single-players and non-players. In addition, children who were multi-players were more likely to have mobile gaming addictions, have mobile violence exposure, experience cyber aggression/victimization, and suffer from emotional and behavioral problems. Children’s mobile gaming addictions were associated with greater risks of exposure to mobile violence/pornography, cyber aggression/victimization, and emotional and behavioral problems. Multi-players, playing violent games, mobile gaming addictions, mobile violence/pornography, cyber aggression/victimization, and poor parent-child relationships were associated with emotional and behavioral problems.
