# Correction: Children’s mobile-gaming preferences, online risks, and mental health

**DOI:** 10.1371/journal.pone.0308315

**Published:** 2024-07-31

**Authors:** Chun-Yin Hou, Ru Rutherford, Hsi Chang, Fong-Ching Chang, Liu Shumei, Chiung-Hui Chiu, Ping-Hung Chen, Jeng-Tung Chiang, Nae-Fang Miao, Hung-Yi Chuang, Chie-Chien Tseng

An additional affiliation is missing for the first author. Chun-Yin Hou is also affiliated with the Department of Health Promotion and Health Education, National Taiwan Normal University, Taipei, Taiwan.
